# The impact of marine recreational fishing on key fish stocks in European waters

**DOI:** 10.1371/journal.pone.0201666

**Published:** 2018-09-12

**Authors:** Zachary Radford, Kieran Hyder, Lucía Zarauz, Estanis Mugerza, Keno Ferter, Raul Prellezo, Harry Vincent Strehlow, Bryony Townhill, Wolf-Christian Lewin, Marc Simon Weltersbach

**Affiliations:** 1 Centre for Environment, Fisheries & Aquaculture Science, Lowestoft, United Kingdom; 2 School of Environmental Sciences, University of East Anglia, Norwich, United Kingdom; 3 AZTI-Tecnalia, Sukarrieta, Spain; 4 Institute of Marine Research, Bergen, Norway; 5 Thünen Institute of Baltic Sea Fisheries (Thünen-OF), Rostock, Germany; Universita degli Studi di Bari Aldo Moro, ITALY

## Abstract

Marine recreational fishing (MRF) has been shown to substantially contribute to fishing mortality of marine fish. However, European MRF catches are only quantified for a small number of stocks, so it is unclear whether a significant part of fishing mortality is excluded from stock assessments. This study estimated: (i) European MRF removals, which were defined as landings plus dead releases; and (ii) impact at stock level by comparing the percentage contribution to total removal by MRF and commercial fishing. As MRF data were limited for some European countries, catches were reconstructed using a mixture of average release proportions, average fish weights, and extrapolation using the catch per fisher of the nearest country providing catch estimates. Where catch reconstructions exceeded 50%, data were excluded from further analysis. Furthermore, as MRF survey methodology can be variable, semi-quantitative estimates of bias and error were calculated for each stock. Only 10 of the 20 stocks assessed in this study had sufficient MRF data for full reliable estimates. Percentage contribution to total removals (MRF + commercial removals) by MRF ranged between 2% for Atlantic mackerel in the North Sea and Skagerrak and 43% for Atlantic pollack in the Celtic Seas and English Channel. The biomass removed ranged between 297 (± 116) tonnes (Atlantic cod in the western English Channel and southern Celtic seas) and 4820 (± 1889) tonnes (Atlantic mackerel in the North Sea and Skagerrak), but the errors were substantial. Additionally, the bias in the estimated removals was low for most stocks, with some positive biases found. The present study indicates that removals by MRF can represent a high proportion of the total removals for some European marine fish stocks, so inclusion in stock assessments should be routine. To achieve this, regular surveys of MRF are required to collect data essential for stock assessments.

## 1.0 Introduction

The spawning stock biomass (SSB) of many commercially important marine fish stocks, such as Atlantic cod (*Gadus morhua*) and European sea bass (*Dicentrarchus labrax*), have declined in recent decades [1,2]. Traditionally, the consensus amongst policy makers and the scientific community has been to attribute the observed declines to commercial fishing pressure and environmental conditions [3–6]. However, the importance of marine recreational fishing (MRF) in biomass removal is becoming more recognised (e.g. [7–13]) leading to inclusion in stock assessment and separate quota allocations for MRF and commerical fishing [14]. For the purposes of this paper, MRF has been defined using the ICES Working Group on Recreational Fisheries Surveys (WGRFS) definition, which is: “*The capture or attempted capture of living aquatic resources mainly for leisure and/or personal consumption*. *This covers active fishing methods including line*, *spear*, *and hand–gathering and passive fishing methods including nets*, *traps*, *pots*, *and set–lines*” [15]. Despite the acknowledgment of MRF as potentially important contributer to fishing mortality, data for many European stocks are limited and do not satisfy end user needs (e.g. stock assessments). The lack of MRF surveys has led to exclusion from assessments in Europe [16], and as a consqeuence, the ability to manage fish stocks sustainably may have been reduced.

Under the EU Data Collection Framework (DCF) [17] European member states have to collect data on recreational landings and releases of Atlantic cod, Atlantic pollack (*Pollachius pollachius*), Atlantic salmon (*Salmo salar*), European sea bass, European eel (*Anguilla anguilla*), sea trout (*Salmo trutta*), elasmobranchs, and ICCAT (International Commission for the Conservation of Atlantic Tunas) species including tuna and tuna-like fishes, with variations in the requirements in different regions. Despite reporting of recreational catches for some species being a requirement since 2002, limited data exist, with few countries carrying out regular surveys. There are a number of potential reasons for this, including the cost of data collection, challenges associated with survey design and implementation, and the belief that MRF has limited impact on marine fish stocks [18]. However, this will change, as pilot studies are required within two years under the latest implementation of the DCF before any derrogation will be granted [17].

Hyder *et al*. [19] found that the participation rate and fishing effort in European marine recreational fisheries can be substantial in both the Atlantic Ocean and Mediterranean Sea, and that European MRF expenditure is just under €6 billion (Table 1). Recent studies relating to the MRF removals of Atlantic cod, European eel, European sea bass, sea trout, and Atlantic salmon stocks have led to MRF being incorporated in assessments of some European fish stocks. The stock assessments that currently include MRF are: European sea bass (ICES divisions 4b&c, 7a,d-h—bss-47 [20,21]), Atlantic salmon in the Baltic Sea (sal-22-31, sal-32) [22], sea trout in the Baltic Sea (trs-22-32) [22] and western Baltic cod (cod-22-24) [23,24]; whilst recreational eel catches are quantified in the EIFAAC/ICES/IGFCM Working Group on Eels (WGEEL) [25] these removals were not considered robust enough to include in a full analytical stock assessment.

**Table 1:** Estimates of numbers, participation, expenditure, and activity by marine recreational fishers in Europe (reproduced from Hyder et al. [19]).

Category	Total
Numbers (millions)	8.67
Participation (%)	1.60
Expenditure (billion €)	5.89
Spend per angler (€)	679
Activity (million days)	77.6

Typically, the literature concerning MRF removals of marine organisms focuses on take of one or more species of fish by a single country (e.g. [10,26–28]). Whilst these data are useful for determining MRF impact on a stock by a single country, the total impact of MRF by all countries on commercially important fish stocks remains unknown. Due to the high MRF effort and catches, the potential impact may be high for some stocks and could introduce additional uncertainty currently not included in stock assessments. In this study, an attempt to estimate the impact of MRF by European countries (including Norway) exploiting marine fish stocks in the Northeast Atlantic (including the North Sea and Baltic Sea), Mediterranean Sea and Black Sea was made. The quality of estimates was assessed using the proportion reconstructed and bias investigated using a simple semi-quantitative score. The results are discussed in the context of fisheries assessment and future monitoring needs.

## 2.0 Material and methods

### 2.1 Data collection

An extensive literature review was conducted to obtain MRF data from existing studies for the Baltic Sea, North Sea, North-Eastern Atlantic Waters, South-Eastern Atlantic Waters, Mediterranean Sea and Black Sea. As a considerable proportion of fish caught by MRF can be released [11,29,30], estimates of post-release mortalities were compiled to determine the total MRF removal (landings plus fish that died post release). Where suitable recreational post-release mortality estimates could not be identified, a precautionary mortality rate of 100% was used. Data were compiled by stock for the key recreational species covered by the DCF for each of the six marine regions. This involved building on the approach developed by Hyder *et al*. [19] and the use of landings and releases by MRF in each country collated annually by WGRFS [31].

Assessing the impact of MRF on European fish stocks proved to be challenging as not all countries conduct surveys and, where surveys were done, the focus was often on one or two key species. Thus, extrapolations were conducted to determine the impact of MRF in countries where data were not available. In the interests of reporting MRF impact at a regional scale, stocks with large boundaries (such as mackerel in the Northeast Atlantic) were split into smaller regions, which were often pre-defined by the ICES working groups responsible for the stock assessment (e.g. WGWIDE, WGEEL).

Commercial landings data for each country were taken from ICES stock assessments for comparisons of commercial and recreational catches. As most MRF data were from 2012, this was used as a reference year and commercial data also selected from 2012. Though, the latest recreational and commercial figures were used where the ICES stock assessment included estimates of MRF catches.

### 2.2 Reconstructions of marine recreational catches

MRF catches were not available for all countries that can potentially exploit each stock, so it was necessary to reconstruct catches to assess the total biomass removed and make a comparison with commercial fishing. As each country had different data available, several different approaches were needed, which are described in detail below.

To calculate the total MRF catch for each stock (*C*_*R*_) it was necessary to add the tonnages kept (*K*_*i*_) and released tonnage that died (∂*R*_*i*_) for each country (*i*), where *m* countries exploit the stock. This was done using the following equation:

$$C_{R}=\sum_{i=1}^{m}\left(K_{i}+\partial R_{i}\right)$$ (1)

where ∂ is the post-release mortality rate and *R*_*i*_ is the weight of released fish in tonnes. Recreational post-release mortality rates would usually be applied to the numbers of fish released, but this made no difference as the average weight was used to derive numbers that was constant across all countries within a stock. There were three ways of obtaining kept weight for each country depending on the data available, these methods were applied in order from the top of Eq 2 down, as this represented less robust solutions and increased uncertainty. Where kept weight (*K*_*i*_) was available, it was used directly, but in many cases only the number of fish kept were available, thus, kept weight was derived from numbers kept (*k*_*i*_) multiplied by the average weight of a kept fish across the whole stock ($\bar{w_{k}}$). Where no data were available, it was necessary to extrapolate using the number of fish kept per fisher from a donor country (*Θ*_*j*_), which was selected based on geographical proximity and similarity of fishing practices to the recipient country, along with the number of fishers from the recipient country (*n*_*i*_). This calculation was done using the following equation:

$$K_{i}=\left\{ \begin{array}{l} K_{i}\;(weight\;kept)\\ k_{i}\bar{w_{k}}\;(number\;kept)\\ \Theta_{j}n_{i}\bar{w_{k}}\;(no\;data) \end{array}\right.$$ (2)

The calculation of released weight was similar to kept weight, but four calculations were required. Again, these were applied in order from the top of Eq 3 down as this represented a less robust solution and increased uncertainty. Where the weight of released fish (*R*_*i*_) was available, it was used in the calculation. If the number released (*r*_*i*_) was available for the country (*i*), then this was multiplied by the average weight of a released fish for the whole stock ($\bar{w_{r}}$). If only the kept weight existed, then released weight was derived from the average proportion released across the whole stock (*ρ*), which was calculated by dividing the sum of all countries’ released numbers where the retained catch was also available by the sum of the retained plus released numbers where both were available, the numbers kept (*K*_*i*_), and the average weight ($\bar{w_{r}}$). Finally, where there were no data, it was necessary to extrapolate using the number of fish released per fisher from a donor country (*Ψ*_*j*_) along with the number of fishers from the recipient country (*n*_*i*_). This calculation was conducted using the following equation:

$$R_{i}=\left\{ \begin{array}{l} R_{i}\;(weight\;released)\\ r_{i}\bar{w_{r}}\;(number\;released)\\ {Κ}_{i}\rho \bar{w_{r}}\;(number\;kept)\\ \Psi_{j}n_{i}\bar{w_{r}}\;(no\;data) \end{array}\right.$$ (3)

To estimate the tonnage of fish released that die it was necessary to assume that recreational release proportion and mortality and commercial discard proportion and mortality were the same across countries, stocks and fishing gear. This is unlikely to be the case as fishing gears and practices vary between countries, but limited experimental data exist. As different gears are often employed by recreational fishers, each of which will have a different post-release mortality, a catch breakdown by individual gear was required to estimate the dead releases, but this was often not available. Consequently, the recreational post-release mortality used was that associated with the highest yielding gear type where data were available, which was rod and line in every circumstance for MRF. The commercial discard mortalities applied were selected as: representative of the fishing fleet or used in the ICES stock assessment. Where extrapolations of catch per fisher were made between countries, the implicit assumption was that fishers in the recipient country fish in the same way and catch the same amount as in the donor country. In addition, average weights of individual fish were assumed to be constant within each stock. For countries that exploited multiple stocks of the same species (e.g. French exploitation of sea bass), it was necessary to assume that CPUE was uniform across the country as no other information existed.

As reconstructions introduce additional uncertainty into the catch estimates, a threshold level was set above which reconstructions were not valid and were not used. This threshold was set at 50% for the landed plus released catch in weight. Thus, if the percent of the total catch reconstructed was below 50% then total catches were considered to be reliable. In the case of sea bass in ICES divisions 8c and 9a (southern Bay of Biscay and Atlantic Iberian waters) the reconstruction percentage was not considered as the data were raised to national level from regional scale studies.

### 2.3 Bias estimation

The scope and methods used by recreational fisheries surveys can differ (e.g. some studies do not sample all platforms such as shore, boat, or spearfishing), so an assessment of the potential bias in the estimates was conducted using the methods described in [19,32]. This involved assigning each study a bias value on a seven-point scale (ranging from -3, denoting highly underestimated, to +3, denoting highly overestimated) to determine the magnitude and direction of bias for each study (*b*_*i*_). All potential sources of bias listed in [19,33,34] were considered. Each study bias was weighted (*w*_*i*_) by the contribution to the total tonnage removed, to reduce the impact of large bias in small estimates. Hence, to calculate the relative bias in each stock *s* (*B*_*s*_) the following equation was used:

$$B_{S}=\sum_{i=1}^{m}b_{i}w_{i}/\sum_{i=1}^{m}w_{i}$$ (4)

where *w*_*i*_ and *b*_*i*_ was assumed to be the same for the donor and recipient countries. The relative biases for each stock were subsequently assigned to a categorical scale (±0.2 ≤ minimal < ±0.4; ±0.8 ≤ moderate < ±1.6; and ±1.6 ≤ large).

### 2.4 Calculation of error bounds

As raw MRF catch data were not available for use in this study, the upper and lower error bounds around the total removals had to be estimated. An attempt to combine the errors in MRF surveys were made, however, error estimates were not available for several countries’ estimates; additionally, the additional error induced when splitting the catches reported by a study to stock level could not be determined. For the two stocks where all errors were identified the coefficient of variation (CV) was 20%, which is also the maximum regional CV allowed by the DCF [35]. Therefore, a 20% CV in the removal estimates was assumed for all stocks when calculating the error bounds.

### 2.5 Comparison of recreational and commercial catches

Usually an assessment of fishing impact would be done through a full analytical stock assessment that includes both recreational and commercial catches. However, this was not possible with the available data or number of species and was not within the scope of the present study. Instead, a simple approach was used to quantify the potential impact of MRF, where the relative contribution of commercial and recreational exploitation of the stock was used. Comparisons were generally made in terms of the biomass removed by commercial (landings plus dead discards) and recreational (landings plus dead releases) fishing. However, the comparison for salmon was based on numbers of fish caught and released in order to be comparable with the stock assessment.

## 3.0 Results

### 3.1 Data compilation and selection

Data were compiled for Atlantic cod, European eel, Atlantic mackerel, Atlantic pollack, Atlantic salmon, European sea bass, sea trout and tuna stocks. Out of the 20 stocks assessed, sufficient data for full reliable estimates of MRF removal were only available for ten stocks (Table 2). Recreational release proportions, which ranged between 0.10 and 0.67, were greater than commercial discarding rates in all stocks except for cod in the Western English Channel and Southern Celtic Seas (Table 3; S2 Table), where the rates were similar. The majority of commercial discard mortalities (Table 4) were precautionary set at 100%, whereas recreational post-release morality studies were available for all but four species.

**Table 2:** The percentage of recreational catch (landings + releases) weight reconstructed for each stock. NA values indicate there are no data to reconstruct landings.

Species	Stock	Area	Total catch reconstructed (%)
Cod	cod-22-24	Western Baltic Sea	0
	**cod-2532**	**Eastern Baltic Sea**	**76**
	cod-347d	North Sea, Eastern English Channel, Skagerrak	17
	cod-7e-k	Western English Channel and Southern Celtic Seas	8
Eel*	**ele-3a,4,7**	**North Sea, English Channel, Skagerrak**	**65**
	**ele-balti**	**Baltic Sea**	**51**
Mackerel*	**mac-1,2,5,14**	**Eastern Arctic**	**NA**
	mac-34	North Sea and Skagerrak	43
	**mac-6**	**West of Scotland**	**NA**
	mac-7,8abde	Celtic seas and Northern and central Bay of Biscay	12
	**mac-8c9a**	**Southern Bay of Biscay and Atlantic Iberian waters**	**66**
Pollack	pol.27.67	Celtic Seas and the English Channel	19
	**pol-89a**	Bay of Biscay and Atlantic Iberian waters	**77**
	**pol-nsea**	**North Sea**	**NA**
Salmon	sal-22-31	Baltic Sea excluding the Gulf of Finland	16
Sea bass	bss-47	Central and southern North Sea, Irish Sea, English Channel, Bristol Channel, and Celtic Sea	6
	bss-8ab	Northern and central Bay of Biscay	0
	bss-8c9a**	**Southern Bay of Biscay and Atlantic Iberian waters**	**61**
**Sea trout**	**trs-22-32**	**Baltic Sea**	**92**
Tuna	**tun-nea**	**Northeast Atlantic**	**NA**

Bold values are above the 50% reconstructed weight cut off.

* = ICES stock assessment based on entire Northeast Atlantic stock and so have been split into sub stocks for this study.

** = Despite having above 50% total catch reconstructed the catches were determined to be representative of the total catch due to the data being raised from small scale national studies.

**Table 3:** Recreational release proportions for each stock analysed in this study. Proportions were calculated by dividing the releases by the catches (landings + releases). Recreational proportions were an average of all studies.

Species	Stock	Recreational release probability
Cod	**cod-22-24**	**0.32**
	**cod-2532**	**NA**
	cod-347d	0.25
	cod-7e-k	0.10
Eel	ele-3a,4,7	0.53
	ele-balti	0.28
Mackerel	mac-1,2,5,14	NA
	mac-34	0.14
	mac-6	NA
	mac-7,8abde	0.24
	mac-8c9a	NA
Pollack	pol.27.67	0.67
	pol-89a	NA
	pol-nsea	NA
Salmon	**sal-22-31**	**0.30**
Sea bass	bss-47	0.62
	bss-8ab	0.50
	bss-8c9a	0.57
Sea trout	**trs-22-32**	**NA**
Tuna	tun-nea	NA

Bolded rows indicate where 2015 data were used rather than 2012 data.

**Table 4:** Recreational post-release mortality and commercial discard mortality rates (%) used to estimate the quantity of dead releases/discards. A precautionary value of 100% was set when no post-release mortality or discard mortality data were available in the literature or the stock assessment for this species for marine environments.

		Recreational		Commercial	
Species	Areas	%	Source	%	Source
Atlantic cod	Atlantic	16.5	[36]	32.0	[37]
Atlantic cod	Baltic	11.2	[38]	32.0	[37]
Atlantic mackerel	All	100.0	Precautionary value	100.0	Precautionary value
Atlantic pollack	All	100.0	Precautionary value	100.0	Precautionary value
Atlantic salmon	All	25.0	[22]	100.0	Precautionary value
European eel	All	100.0	Precautionary value	100.0	Precautionary value
European sea bass	All	5.0	[39]	100.0	Precautionary value
Sea trout	All	100.0	Precautionary value	100.0	Precautionary value
Tuna	All	5.6	[40]	100.0	Precautionary value

### 3.2 Marine recreational and commercial fishing removals

**Fig 1:**
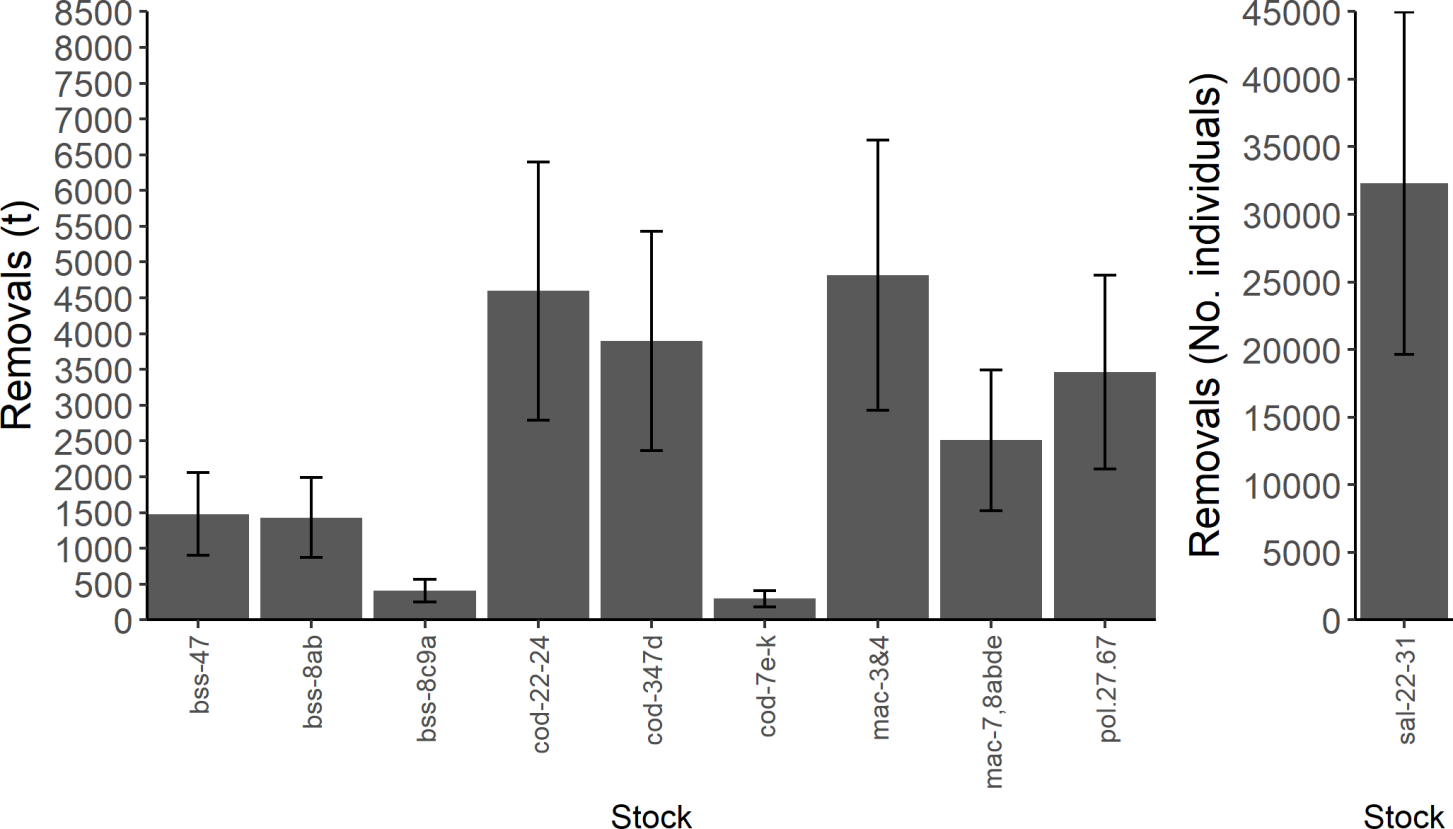
Total removals (± error bounds) by MRF from each stock. The total estimated removals (biomass in tonnes or number of individuals landed + post-release mortality) from each stock (± confidence bounds) by MRF. Sal-22-31 removals were presented on a separate scale due to the use of number of individuals removed rather than weight, further, sal-22-31 represents marine catches only. See S8 Table for raw data.

**Fig 2:**
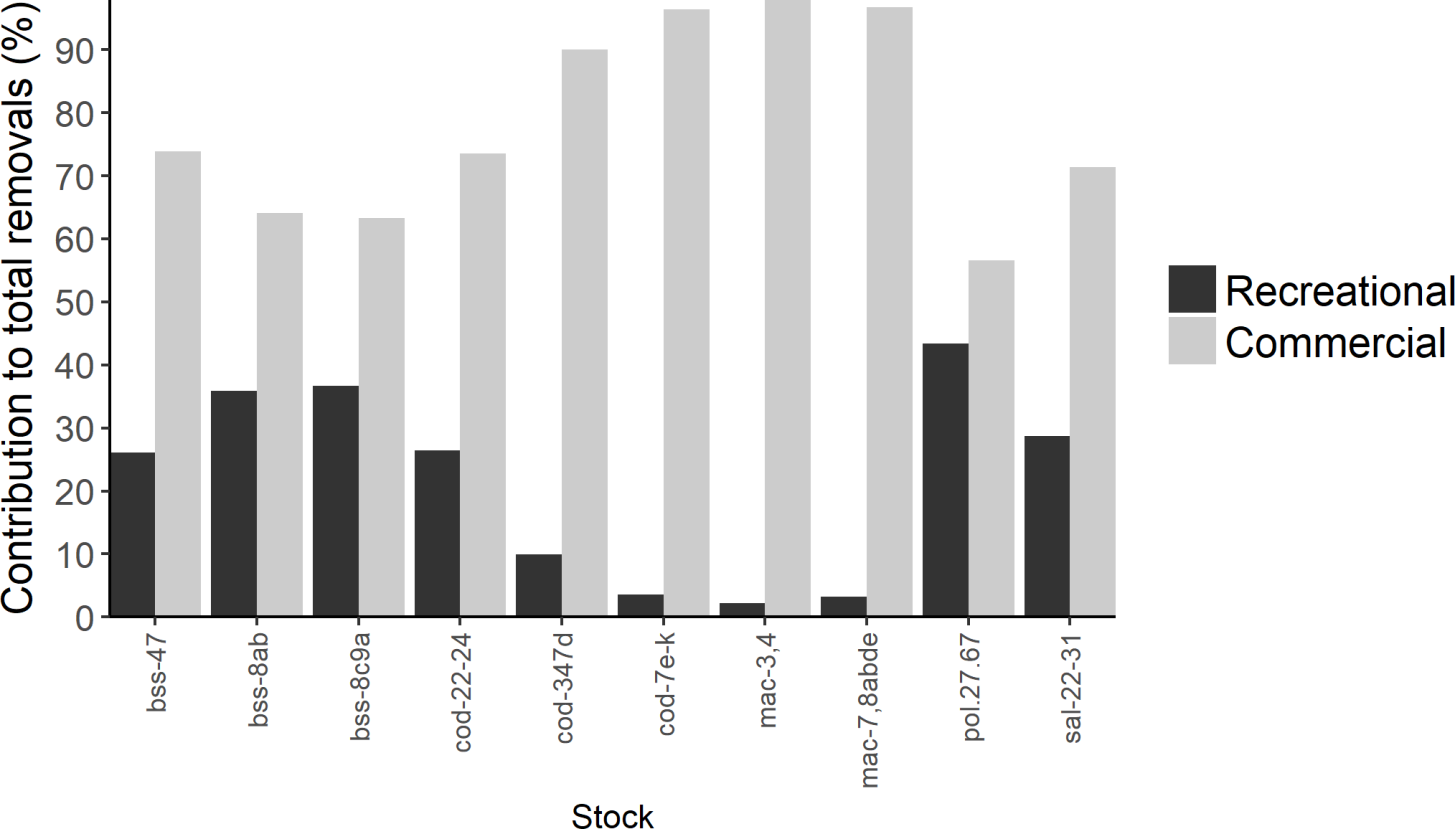
Contribution to total removals by recreational and commercial fishing. The percentage contribution to total removals (= landings/ (landed + dead releases or discards)) by recreational and commercial fishing for each stock in which sufficient data existed examined in this study. Salmon removals did not include freshwater recreational fishing data as this study focused on marine recreational fishing.

MRF biomass removals were substantial in almost all stocks (Fig 1; Table 2; S1–S7 Tables), particularly western Baltic cod (cod-22-24 4595t ±1801t; S2 Table), North Sea, Eastern English Channel and Skagerrak cod (cod-347d 3899t ±1528t; S2 Table), all Atlantic mackerel (4820t ± 1889t and 2510t ± 984t for mackerel in the North Sea and Skagerrak, mac-3,4, and in the Celtic Sea, mac-7,8abde, respectively; S3 Table) and Atlantic salmon stocks (32290 ±12658 individuals) in the Baltic Sea (Fig 1; S6 Table). However, when compared with the commercial biomass removal, the contribution to the total biomass removed by MRF was minimal for several species, such as North Sea, Eastern English Channel and Skagerrak cod (cod-347d), for which MRF accounts for only 10% of total removals (Fig 2; S2 Table). Conversely, the removal of Atlantic pollack biomass in the Celtic Seas and English Channel (pol-27.67) by MRF was similar to the commercial fishing fleets (43% of total removals); additionally, MRF removals for cod in the western Baltic Sea (cod-22-24) were considerable when compared to the commercial fleet (26% of total removals).

### 3.3 Bias estimation

**Fig 3:**
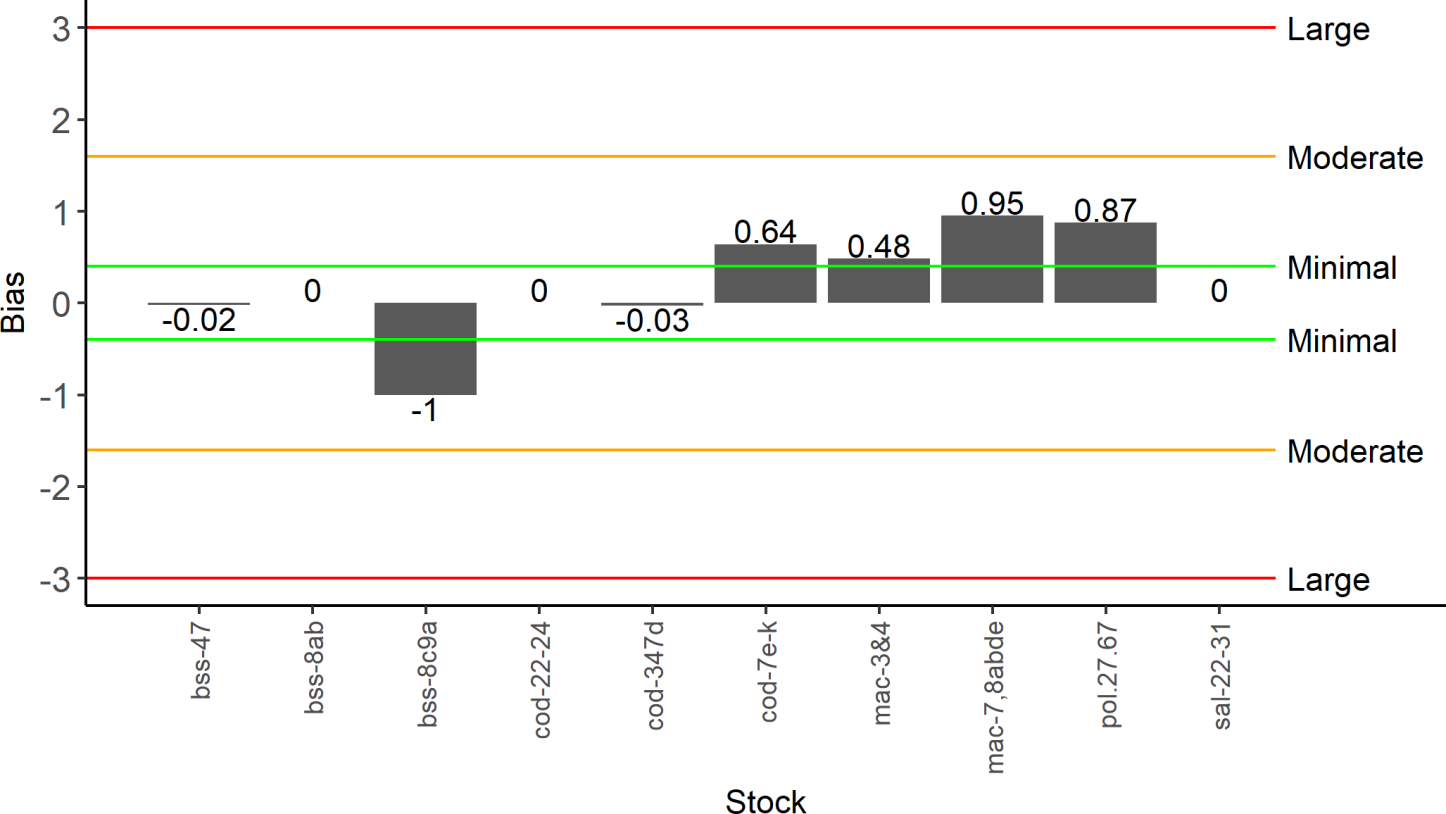
Estimated recreational removal bias for each stock. The estimated bias in the total MRF removals (landings + post-release mortality) for each stock calculated using a weighted study bias. Bias was rated on a seven-point scale ranging between +3 denoting highly overestimated and -3 denoting highly underestimated.

Overall, the level of bias in all the studies used was low (Fig 2). The level of bias in the estimate of total MRF removals at stock level was generally very low, with five stocks having minimal to no bias in the catch estimates (Fig 3). Where biases were found, moderate overestimation in the results was the most common with only sea bass in the southern Bay of Biscay and Atlantic Iberian waters (bss-8c9a) being moderately underestimated (Fig 3; S1 Table). Further, no discernible bias was found for western Baltic cod (cod-22-24), Northern and central Bay of Biscay sea bass and Baltic salmon (sal-22-31) (Fig 3; S2 Table).

### 4.0 Discussion

#### 4.1 Comparison of MRF and commercial fishing catch proportions

The total impact of MRF on fish stocks was found to be substantial in several European stocks. Even in those stocks in which the percentage contribution to total landings were low, the total biomass removal by MRF was in some cases greater than the national catches by commercial fishing (e.g. mac-34 recreational catches were greater than Dutch and Belgian commercial landings despite MRF only contributing to 2% of the total removals; S3 Table). The large removals by MRF in many stocks could be impacting the ability to manage fish stocks sustainably as this biomass removal is not accounted for in many cases when providing advice regarding the total allowable catch (TAC) of a stock. MRF catches were examined in an international context by Hyder *et al*. [19], who found that the MRF removals of western Baltic cod (ICES subdivision 22–24) and sea bass in ICES division areas IVb-c and VIIa, d-h (bss-47) were high (4679 tonnes and 1468 tonnes, or 26% of total removals for both stocks, respectively). The MRF catches reported by Hyder *et al*. [19] were similar to those found in this study but not identical. This is due to the current study including MRF and commercial data from several other countries that were not considered by Hyder et al. [19] as they did not reconstruct missing data. The almost identical percentage removals by MRF found in this study and Hyder et al. [19] despite the difference in methodologies provides further evidence for the potential for MRF to remove a significant biomass from a stock.

The percentage removal by MRF was lower than by commercial fishing for all stocks. The stocks of sea bass in the North Sea, Irish Sea, English & Bristol Channel, and Celtic Sea (1480t or 26% of total removals S1 Table), cod in the western Baltic Sea (4595t or 26% of total removals), salmon in the Baltic Sea (32290 individuals or 29% of removals) and Pollack in the Celtic Seas and English Channel (3,463t or 43% of total removals S5 Table) were found to have the highest percentage contribution to total landings by MRF. However, the high MRF catches calculated for pollack in the Celtic Seas and English Channel were driven by high recreational landings in France (as estimated by Herfaut *et al*. [26]), though, this study may have overestimated French recreational catches. Nevertheless, the findings of this study and several others (e.g. [36,27,28,41,42]) have illustrated that MRF impact on sea bass stocks is significant, which has contributed to the EU’s Scientific, Technical and Economic Committee for Fisheries decision to call for an 80% reduction in total sea bass landings [43]. Cooke & Cowx [8] also compared the percentage contribution to total removals by recreational and commercial fishing within the USA, finding significant proportions (17%) of cod catches were due to MRF. Whilst the proportion of Atlantic cod removed by MRF in the USA is greater than two of the stocks quantified in this study (cod-347d: 10%, cod-7e-k: 4%; S2 Table), this is probably due to the greater number of recreational fishers exploiting fish stocks in the USA [44,45] compared to Europe [19]. In Norway, Kleiven *et al*. [46] found considerably greater recreational rod and line fishing mortality rates for Atlantic cod (34%) than was found in this study. However, this was based off a tag and release study of an inshore cod population rather [46] than comparing the total tonnage removed by all countries explointing the stock, as was done in the present study, which may account for the difference in fishing mortality.

This study also identified a large variation in the proportion of total catches by MRF between species. Variations in MRF catches may be attributed to the accessibility of a stock and the popularity of certain species (e.g. due to its catchability, size, fighting ability, or culinary value) for anglers [47]. Therefore, populations that are situated or migrate inshore may be at greater risk of high exploitation if the MRF intensity is substantial. However, if the biomass of the stock is above the lowest acceptable limit (B_lim_), then the impact of MRF alone is unlikely to impact on sustainability. If other factors (e.g. poor recruitment years) were to reduce the stock biomass, then MRF removals could put considerable pressure on the stock.

### 4.2 Potential error and bias in the data

All the data used in the present study were taken from other studies, so the reliability of the estimates was evaluated prior to interpreting the results. Several assumptions were made when extrapolating the data and applying post-release mortalities introducing additional uncertainty into the estimates. However, extrapolation percentages where full and partial comparisons were made were generally low except for sea bass in the Southern Bay of Biscay and Atlantic Iberian waters (bss-8c9a) and therefore the impact of reconstructions were minimal. For sea bass in the Southern Bay of Biscay and Atlantic Iberian waters, the MRF catches were raised from small-scale national studies [48] (DCF Sampling, unpublished data), which reduced the risk of bias of MRF removals and was therefore likely to be representative of the true catch. A large error bound relative to the total removals was calculated for all stocks (S8 Table), the error bound calculation assumed a constant CV of 20%, which is the maximum allowed in DCF studies [35], and may therefore generate different estimates of error for total MRF removals.

Another potential source of error in the results was the post-release mortalities used for both recreational and commercial releases/discards, as in most cases country-specfic differences in fishing practices (e.g gear type used) could not be taken into account. Additionally, where post-release mortalities could not be determined, a precautionary 100% mortality rate was used. A full description of the factors affecting post-release mortality is not provided here, but see Bartholomew & Bohnsack [30] for examples of the factors impacting recreational angling post-release mortalities for various species and Alverson, *et al*. [49] for examples of commercial fishing discard mortality. Whilst commercial discard mortality and recreational post-release mortality can be very high for some species, the precautionary 100% post-release mortality rate applied, where data were not readily available, is most likely greater than the actual post-release mortality that occurs, which may induce overestimation of removals by both MRF and commercial fishing. Conversely, using mortality values derived from studies conducted under certain circumstances for discards by commercial fishing or releases by MRF may result in underestimation of catches as country-specific differences in fishing practices will cause different associated mortality rates (e.g. [39, 49]). The impact of not taking into account gear type in MRF post-release mortality was likely to be larger in countries allowing static gears (e.g. gill nets) for recreational use, such as Sweden [50], as these gears are likely to have significantly higher post-release mortality than angling. However, as angling was the predominant method of MRF found in all studies that quantified percentage contribution to total MRF landings (e.g. [41,50]), the impact of applying post-release mortality estimates from angling is minimal.

In terms of overall bias in stock-level removal estimates, sea bass in the southern Bay of Biscay and Atlantic Iberian waters (bss-8c9a) was the only stock identified where a negative bias in the removals calculated may have occurred. This is due to both the total Portuguese and Spanish catch estimates being raised from small-scale national studies [48] (DCF Sampling, unpublished data). Furthermore, whilst no bias in MRF catch estimates was detected on a stock level for western Baltic cod, northern and central Bay of Biscay sea bass and Baltic salmon, bias within an individual country MRF catch estimate as well as that introduced by reconstructing data may still have occurred. Additionally, it is worth noting that the method used to quantify bias may not have been sensitive or robust enough to detect all forms of bias in the catch estimates. The issues with the estimation of bias in this study were due to the quantification of the bias for each study, in addition to the difficulty in measuring bias introduced by reconstructing data. However, as the intention was to indicate the potential magnitude and direction of bias rather than estimate the number or weight of fish that were over/underreported by each study the impact of bias estimation issues were minimal.

Due to the potential for uncertainty and bias in the data, the results of this study should only be used as an indicator of the impact of MRF in European marine fish stocks and should not be used as a replacement for national recreational fisheries sampling schemes. Furthermore, time series of MRF catches show large variation in catch-per-unit-effort and catches between years [10,23,24], which is not captured in this study. These variations underline the importance to collect annual estimates of catches for inclusion in stock assessments otherwise assumptions are required to generate time series from data from either a single year, such as in the sea bass assessment [20,21], or to deal with intermittent data.

### 4.3 Data limitations

#### 4.3.1 Data poor species

MRF data for both elasmobranch and tuna species were extremely limited despite being listed as key species in the DCF, with only Senegal reporting recreational tuna catches in the ICCAT database [51] and MRF catches for many elasmobranch species were aggregated inconsistently in reports. The lack of data for both tuna and elasmobranch species meant that no attempt to reconstruct recreational removals or compare recreational and commercial removals could be made.

#### 4.3.2 Data poor regions

Despite the potential impact of MRF on marine fish stocks, as revealed by this study and several others (e.g. [10,26,28,41,50,52]) and the DCF requirement to report MRF catches [18], some European countries do not currently provide estimates of MRF catches for use in stock assessments, which can severely impact the advice provided by stock assessments. Furthermore, national MRF sampling schemes often vary in methodology and scope. Even though variation in MRF sampling methodology is inevitable due to regional differences between angler behaviour and fishing practices, a universal assessment of quality is needed to ensure that the results can be summed across studies [31].

Although an attempt to characterise fisheries within the Mediterranean and Black Seas has been made as part of the ‘SeasAroundUs’ project (http://www.seaaroundus.org/) [53–58], the landings provided were an average for 2010–2013 and were not split by country. In addition, the MRF catch estimates attained at a country level were reconstructed for almost all species [53–58] meaning further reconstructions would have introduced a large amount of uncertainty, so were unlikely to be representative of the true catch. Indeed, Venturini et al. [59] also quantified MRF catches in an Italian marine protected area, however, the study area was considered too small to reconstruct data from. Consequently, the impact of MRF in the Mediterranean and Black Seas could not be determined in this study. It is essential that additional MRF surveys are to be conducted in order to accurately quantify the impact, particularly in the Mediterranean and Black Seas.

## 5.0 Conclusion

MRF catches were shown to be a significant proportion of the total removals and varied between 2% and 43% by stock. Despite these large removals, MRF has not been considered in the stock assessment for some of the stocks investigated in this study, indicating a potentially large amount of fishing mortality in the stock is unaccounted for. Furthermore, data for MRF catches are limited and, where available, generally only cover species required by DCF. As a result, reconstruction is required to estimate total removals by MRF, which induces increased error in removal estimates. Multispecies surveys would be beneficial as time series are needed for inclusion in stock assessment and the costs of the additional data collection are minimal. It is also worth noting that the comparison made here between the levels of commercial and recreational catches gives an idea of the potential impact of MRF, but inclusion in a full analytical assessment is needed to properly assess the impact due to differences in selectivity.

## Supporting information

S1 Table Bass data reconstruction. (XLSX)

S2 Table Cod data reconstruction. (XLSX)

S3 Table Mackerel data reconstruction. (XLSX)

S4 Table Eel data reconstruction. (XLSX)

S5 Table Pollack data reconstruction. (XLSX)

S6 Table Salmon data reconstruction. (XLSX)

S7 Table Sea trout data reconstruction. (XLSX)

S8 Table Error estimation Table. (XLSX)

S1 Text Supplementary material references. (DOCX)
