# Supplementary material for: The impact of marine recreational fishing on key fish stocks in European waters
Source: PLoS One. 2018 Sep 12;13(9):e0201666. doi: 10.1371/journal.pone.0201666 (PMC6135385; doi:10.1371/journal.pone.0201666)
Supplement: S1 Text — (DOCX) [file pone.0201666.s009.docx]

Arkenfold. Watersports participation survey. London, UK; 2016.

Armstrong M, Brown A, Hargreaves J, Hyder K, Munday M, Proctor S, et al. Sea Angling 2012 – a survey of recreational sea angling activity and economic value in England. London, UK; 2013.

Capizzano CW, Mandelman JW, Hoffman WS, Dean MJ, Zemeckis DR, Benoît HP, et al. Estimating and mitigating the discard mortality of Atlantic cod (*Gadus morhua*) in the Gulf of Maine recreational rod-and-reel fishery. ICES J Mar Sci J du Cons. 2016;73: 2342–2355.

Depestele J, Desender M, Benoît HP, Polet H, Vincx M. Short-term survival of discarded target fish and non-target invertebrate species in the “eurocutter” beam trawl fishery of the southern North Sea. Fish Res. 2014;154: 82–92.

Ferter K, Weltersbach MS, Strehlow H V, Vølstad JH, Alós J, Arlinghaus R, et al. Unexpectedly high catch-and-Release rates in European marine recreational fisheries. ICES J Mar Sci. 2013;70: 1319–1329.

Herfaut J, Levrel H, Thébaud O, Véron G. The nationwide assessment of marine recreational fishing: A French example. Ocean Coast Manag. 2013;78: 121–131.

Hyder K, Weltersbach MS, Armstrong M, Ferter K, Townhill B, Ahvonen A, et al. Recreational sea fishing in Europe in a global context-Participation rates, fishing effort, expenditure, and implications for monitoring and assessment. Fish Fish. 2018;19: 225–243.

ICES. Catch statistics [Internet]. 2017. Available: http://www.ices.dk/marine-data/dataset-collections/Pages/Fish-catch-and-stock-assessment.aspx

Insee. Estimation de la population au 1er janvier 2018 [Internet]. 2017 [cited 6 Jun 2017]. Available: https://www.insee.fr/fr/statistiques/1893198

Karlsson M, Ragnarsson Stabo H, Petersson E, Carlstrand H, Thörnqvist S. A national data collection framework for recreational fishing. Drottningholm, Sweden; 2016.

Lewin W-C, Strehlow H V, Ferter K, Hyder K, Niemax J, Herrmann J-P, et al. Estimating post-release mortality of European sea bass based on experimental angling. ICES J Mar Sci. 2017; doi:10.1093/icesjms/fsx240

MMO. UK fleet landings by ICES rectangle [Internet]. 2016 [cited 20 May 2017]. Available: https://www.gov.uk/government/collections/uk-sea-fisheries-annual-statistics

Persoon K. Who is the recreational fisherman and what does he catch? An overview of recreational fisheries at sea in Belgium. Instituut voor Landbouw-en Visserijonderzoek. MSc. Thesis. 2015; 76pp.

Rangel M, Erzini K. An assessment of catches and harvest of recreational shore angling in the north of Portugal. Fish Manag Ecol. 2007;14: 343–352.

Rocklin D, Levrel H, Drogou M, Herfaut J, Veron G. Combining Telephone Surveys and Fishing Catches Self-Report: The French Sea Bass Recreational Fishery Assessment. Stergiou KI, editor. PLoS One. 2014;9: e87271.

Sparrevohn CR, Storr-Paulsen M. Using interview-based recall surveys to estimate cod Gadus morhua and eel Anguilla anguilla harvest in Danish recreational fishing. ICES J Mar Sci. 2012;69: 323–330.

Strehlow H V., Schultz N, Zimmermann C, Hammer C. Cod catches taken by the German recreational fishery in the western Baltic Sea, 2005-2010: implications for stock assessment and management. ICES J Mar Sci. 2012;69: 1769–1780.

van der Hammen T, de Graaf M. Recreational fishery in the Netherlands: demographics and catch estimates in marine and fresh water. 2013; 33.

van der Hammen T, de Graaf M. Recreational fisheries in the Netherlands: analyses of the 2012-2013 online logbook survey, 2013 online screening survey and 2013 random digit dialling screening survey. CVO report: C042/15, Wageningen, Netherlands; 2015.

van der Hammen T, de Graaf M, Lyle JM. Estimating catches of marine and freshwater recreational fisheries in the Netherlands using an online panel survey. ICES J Mar Sci. 2016;73: 441–450.

Veiga P, Ribeiro J, Gonçalves JMS, Erzini K. Quantifying recreational shore angling catch and harvest in southern Portugal (north-east Atlantic Ocean): implications for conservation and integrated fisheries management. J Fish Biol. 2010;76: 2216–2237.

Vølstad JH, Korsnrekke K, Nedreaas KH, Nilsen M, Nilsson GN, Pennington M, et al. Probability-based surveying using self-sampling to estimate catch and effort in Norway’s coastal tourist fishery. ICES J Mar Sci. 2011;68: 1785–1791.

Weltersbach MS, Strehlow H V. Dead or alive—estimating post-release mortality of Atlantic cod in the recreational fishery. ICES J Mar Sci. 2013;70: 864–872.

WGBFAS. Report of the Baltic Fisheries Assessment Working Group (WGBFAS). Copenhagen, Denmark. ICES CM 2017/ACOM:11; 2017.

WGBIE. Report of the Working Group for the Bay of Biscay and Iberian waters Ecoregion (WGBIE). Cadiz, Spain. ICES CM 2017/ACOM:12; 2017.

WGCSE. Report of the Working Group on Celtic Seas Ecoregion (WGCSE). Copenhagen, Denmark. ICES CM 2017/ACOM:13; 2017.

WGNSSK. Report of the Working Group on Assessment of Demersal Stocks in the North Sea and Skagerrak (2017). Copenhagen, Denmark. ICES CM 2017/ACOM:21; 2017.

WGRFS. Report of the Working Group on Recreational Fisheries Surveys (WGRFS). Azores, Portugal. ICES CM 2017/EOSG:20; 2017.

WGWIDE. Report of the Working Group on Widely Distributed Stocks (WGWIDE). Copenhagen, Denmark. ICES CM 2017/ACOM:23; 2017.

World Bank. Population, total. In: United Nations Population Division [Internet]. 2017 [cited 6 Jun 2017]. Available: https://data.worldbank.org/indicator/SP.POP.TOTL

Zarauz L, Prellezo R, Mugerza E, Artetxe I, Roa R, Ibaibarriaga L, et al. Analysis of the recreational and fishing fleet in the Basque Country and its socioeconomic impact. Rev Investig Mar (Marine Res Journal). 2013;20: 38–70.
